# Exploring User Needs in the Development of a Virtual Reality–Based Advanced Life Support Training Platform: Exploratory Usability Study

**DOI:** 10.2196/20797

**Published:** 2020-08-07

**Authors:** Nathan Moore, Soojeong Yoo, Philip Poronnik, Martin Brown, Naseem Ahmadpour

**Affiliations:** 1 Research and Education Network Western Sydney Local Health District Westmead Australia; 2 Design Lab Sydney School of Architecture, Design and Planning The University of Sydney Sydney Australia; 3 School of Medical Sciences Faculty of Medicine and Health The University of Sydney Sydney Australia; 4 Innovative Technologies Office of the Vice-Chancellor and Principal Westmead Operations The University of Sydney Sydney Australia

**Keywords:** virtual reality, Advanced Life Support, Advanced Cardiac Life Support, clinical training, education, serious games

## Abstract

**Background:**

Traditional methods of delivering Advanced Life Support (ALS) training and reaccreditation are resource-intensive and costly. Interactive simulations and gameplay using virtual reality (VR) technology can complement traditional training processes as a cost-effective, engaging, and flexible training tool.

**Objective:**

This exploratory study aimed to determine the specific user needs of clinicians engaging with a new interactive VR ALS simulation (ALS-SimVR) application to inform the ongoing development of such training platforms.

**Methods:**

Semistructured interviews were conducted with experienced clinicians (n=10, median age=40.9 years) following a single playthrough of the application. All clinicians have been directly involved in the delivery of ALS training in both clinical and educational settings (median years of ALS experience=12.4; all had minimal or no VR experience). Interviews were supplemented with an assessment of usability (using heuristic evaluation) and presence.

**Results:**

The ALS-SimVR training app was well received. Thematic analysis of the interviews revealed five main areas of user needs that can inform future design efforts for creating engaging VR training apps: affordances, agency, diverse input modalities, mental models, and advanced roles.

**Conclusions:**

This study was conducted to identify the needs of clinicians engaging with ALS-SimVR. However, our findings revealed broader design considerations that will be crucial in guiding future work in this area. Although aligning the training scenarios with accepted teaching algorithms is important, our findings reveal that improving user experience and engagement requires careful attention to technology-specific issues such as input modalities.

## Introduction

### Background

Advanced Life Support (ALS) training is a mandatory requirement for all medical staff with critical care exposure and all nursing staff are required to attend as part of the ALS team. The term Advanced Cardiac Life Support (ACLS) is synonymous with ALS, with the latter being the generally accepted vernacular in Australia. An ALS team comprises a number of clinicians with a variety of skills and experiences. For such a team to function at the highest level, it is essential that a skilled clinician is identified to take the role of team leader [1]. The role of ALS team leader requires the clinician to manage a patient who is experiencing an acute deterioration or already in cardiac arrest. This management is largely built upon endorsed algorithms such as the Australian Resuscitation Guidelines [2]. These guidelines are typically applied in a highly stressful and chaotic environment where 6 to 10 clinicians must be directed in the tasks required to manage the patient. Unfortunately, many clinicians may be inadequately prepared to do these tasks [3]. The stress and chaos resulting in deviation from the guidelines may lead to catastrophic outcomes for the patient [4].

Currently, ALS training in many hospitals consists of prereading from a manual outlining the procedure algorithm; quizzes with multiple-choice questions; and face-to-face training days including skills stations, didactic sessions, simulations, and a face-to-face assessment. This assessment is then repeated as per institutional requirements, often on an annual basis.

Initial face-to-face training, refresher training, and reaccreditation are resource-intensive processes requiring high instructor-to-participant ratios and costly simulation equipment [5]. Refresher training can pose challenges for educators and clinicians alike [6]. There are logistical challenges common to face-to-face training and reaccreditation, such as getting clinicians with high clinical demands off the hospital floor as well as coordinating recourses and educator time [7].

During face-to-face training, a significant amount of time is spent exploring the technical elements of the ALS algorithm and management, such as drug dosages and timings, rhythm interpretation, defibrillation timings, differential diagnoses, and more. Although these technical elements are important, the exploration and training of them detracts from time that could be spent usefully on the equally important nontechnical elements of ALS management such as communication, teamwork, and situational awareness [8]. These resource challenges have driven a need moving forward to focus on efficient and scalable high-quality health education [9,10].

### Objectives

There is a known and well-represented correlation between reducing the time taken to identify patient deterioration and a marked improvement in patient outcomes in the inpatient setting [11]. As a result, multitiered rapid response systems and mandatory standardized triggers for escalation in patient management have been implemented, leading to earlier identification of patient deterioration [12]. A recent systematic review showed that this early identification has resulted in a pronounced decrease in the number of cardiac arrest calls occurring in inpatient populations [13]. The challenge this presents for clinicians is that cardiac arrest management has now become a low-frequency yet high-stakes event. This in turn means that clinicians have reduced opportunities to perform ALS and consolidate skills. However, when required, they are expected to execute these skills at the highest standard. Even with exposure to these skills in a clinical setting, clinicians require more than practice alone, as further feedback and education are essential [10].

Clinicians and educators must, therefore, explore modalities and technologies that can overcome the barriers of cost, access, and frequency of exposure [14]. Some of the ways of addressing these challenges include simulation-based learning using the latest technologies (eg, virtual reality [VR], which has generated a growing interest in recent years [15]). Studies have demonstrated improved performance when using VR for basic life support training [16], but there is limited information about what users of ALS systems need, specific design considerations, and ways we can improve user experience and engagement with the training [17].

The main objective of this study is to identify key user needs and guidance for designing VR-based educational resources for ALS providers. We achieve this through an exploratory study, using a virtual ALS training module we have created.

### VR Technology for ALS Training

VR offers a number of novel capabilities that have promising potential in providing new kinds of support for educators [18,19]. The ability to visualize and interact with 3D representations in real time and visualize the dynamic relationships between variables in a complex environment [20] makes for a compelling use case in the context of ALS training. In addition, VR technology provides a simulated environment that can help users recreate an experience which would otherwise be difficult to have [21]. Finally, VR provides a unique immersive experience that blocks out the physical world, resulting in the user believing or feeling they are present in the VR environment [22].

These affordances of VR make it an intriguing platform to explore for ALS training. There have been a few attempts at developing similar VR-based training such as Health Scholars ACLS Virtual Reality training [23] or dualgood ACLS and ALS training [24]. However, there are a number of limitations associated with using these platforms in our context, including potential costs as a commercial product and a lack of customization of the training modules, which are based on the American Heart Association guidelines [25] and differ from those endorsed in other countries, such as the Australian Resuscitation Guidelines [26].

In addition to system limitations that prevent the scalability of some current ALS trainings in VR, there is currently a knowledge gap about the perspectives and needs of the end users, who should be included in the development of future applications in this field [27]. A user-centered approach would involve a careful and iterative process that takes into account the training requirements, determinants of positive user experience among trainees, and usability of the system to provide a smooth and engaging interaction in the virtual environment. In this study, we describe and explore this approach in the context of ALS training, and specifically in relation to the most challenging role, that of the ALS team leader.

We identified a few challenges for designing VR to supplement training for ALS team leaders, based on our own experiences of delivering this type of training. One challenge is that participants are often unable to recall information available within the prescribed prereading on the face-to-face training days. The concept of learner fatigue and disengagement when faced with a large amount of prereading is well established [28]. The prereading for this program is a 188-page manual intended as an ongoing resource; however, participants are expected to have a basic understanding of the majority of the concepts contained within prior to the program. The level of immersion in VR has been shown to further facilitate information recall presented in the virtual environment compared to a flat screen [29], upon which the training manual could be viewed. This effect can be amplified through creating a realistic setting in VR to enhance information recall [30]. Furthermore, VR enables the user to explore alternate viewpoints in a given scenario [20]. This could be used to enable the user to see the environment from the viewpoint of other team members as well as to review their own performance from alternate viewpoints.

### Interactive VR Simulations in Medical Education

Interactive virtual simulations have been utilized in medical education to provide students and clinicians engaging ways to learn skills, abilities, and critical knowledge for real-world applications [31,32]. Immersive virtual environments (VE) have been used in the context of health care education to offer development of technical skills in the areas of surgical training [33] and clinical procedures [34], as well as replicating complex team-based systems such as ALS [31]. Until recently, one of the limitations of these VEs has been the need to have high-powered computers, networks, and tethered head-mounted devices (HMD) to provide high-quality VR. The release of the Oculus Quest (Oculus VR), which has 6 degrees of movement and a relatively low unit cost, has allowed for a standalone portable, powerful, and immersive experience.

A notable example of VR-based ALS training simulation is the app developed by Arizona State University [18], which provides a multiplayer collaborative gaming VE. Although there are clear benefits for practicing as a team in a dynamic ALS setting [35], this requires multiple users to be coordinated to practice at the same time in the same VE. This poses a significant logistic and time challenge and does not allow individuals to rehearse on their own time.

In this study, we describe a single-player interactive simulation that overcomes the limitations mentioned above. We developed a prototype VR app that contains elements of interactive simulation, which we then used to study the factors relevant to users’ needs to deliver a positive training experience that is also easy to use. Elements that were of particular importance to the interactivity of the experience include gameplay, a clear purpose, and realistic representation of current environments [36].

### Design

In our experience developing several simulation and flipped-classroom supplemental training assets, any solution to supplement ALS training needs to be flexible and meet the requirements of both the educators and clinicians. We identified two primary usage opportunities that could be of value to the identified audience of an interactive VR-based app and used those to create a frame of reference for prototyping our app, named ALS-SimVR.

The first is the opportunity to use the VR app intermittently in a clinical setting. There is often an overlap period of 30 to 45 minutes during handovers that is traditionally allocated to in-service time. Designing for this opportunity means ensuring that the VR app allows for a complete experience for the user within these time frames. This also implies that the ALS-SimVR app should be deployed on a portable device and require little to no setup time.

The second opportunity is the use of the app for preparation or refreshing knowledge during the user’s private time (eg, at home or in their office). Similarly, this requires portability and an easy setup process.

### ALS-SimVR Application Walkthrough

**Figure 1:**
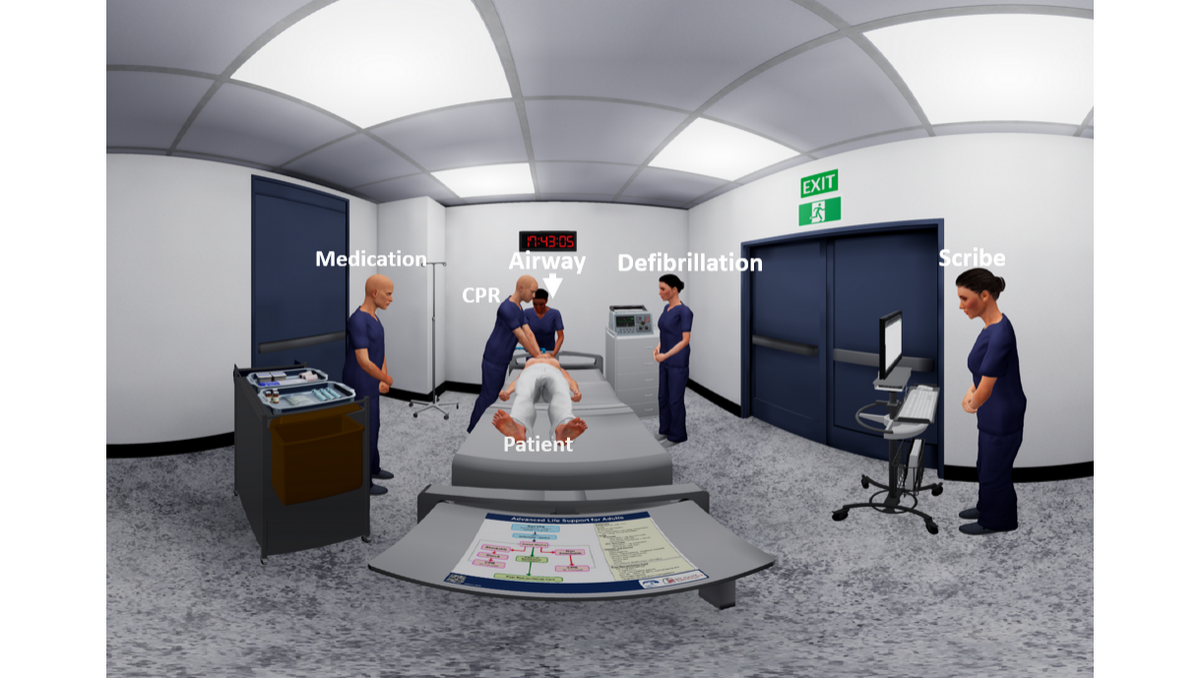
In-game user view displaying the team leader’s perspective.

The interactive simulation places the user in the position of ALS team leader and allows them to direct the team in the management of a patient in cardiac arrest [37]. The virtual space as seen from the team leader’s point of view is shown in Figure 1.

**Figure 2:**
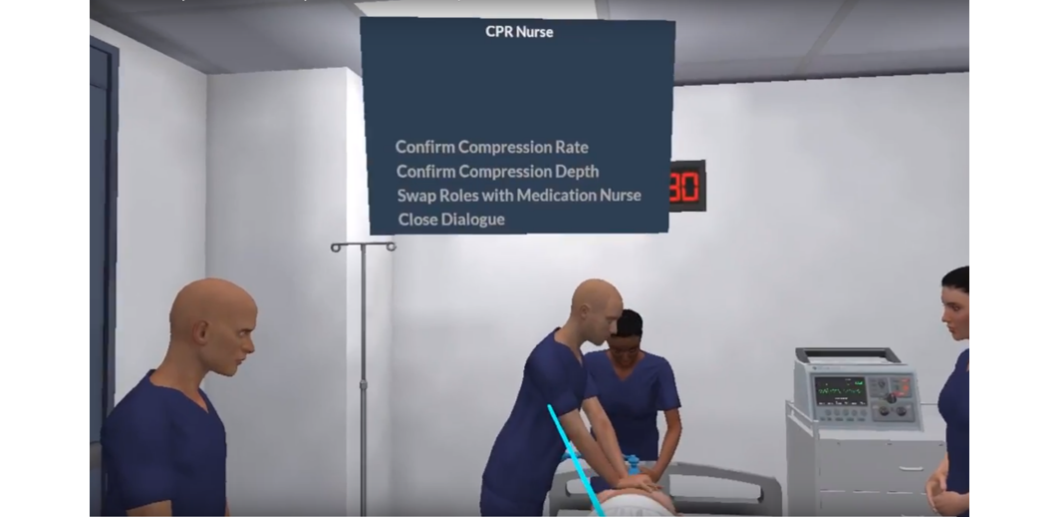
Interactions with virtual team members.

Upon commencement of the scenario, the team leader is presented with a handover from one of the virtual team members, outlining the current clinical situation. The team leader is then free to manage the patient as per their clinical judgement. All management decisions are made by clicking on a virtual team member using the Oculus Go controller via dropdown menus that appear above the virtual team member’s head. These decisions are all recorded and represented on the scribe notes within the application, which are available as a reference for the user. Throughout the scenario, realistic prompts are provided by the virtual team members, such as “difficulty ventilating the patient” or “nearing 2 minutes of CPR,” all of which are generalizable clinical cues which replicate real-world team function (Figure 2).

Vital tasks such as defibrillation, drug and fluid administration, compression/ventilation ratio and rate, role swapping, intravenous access, and airway management are all available as decisions and are replicated realistically in the scenario. The user is free to make decisions at any time they choose, whether the decision is correct or incorrect. Those choices and timings form the assessment provided at the culmination of the scenario.

The scenario within ALS-SimVR mirrors the face-to-face ALS training at Westmead Hospital in New South Wales, Australia. The base scenario was designed in consultation with senior clinicians directly involved in cardiac arrest management and reviewed to realistically replicate a common presentation of cardiac arrest. The scenario mimics pathology and decisions that are generalizable across Australia.

### Overview of the ALS-SimVR App Architecture

The application was created initially for deployment on the Oculus GO. The portable nature of these devices means that clinicians can practice at their own pace prior to attending an ALS program session or annual reaccreditation.

ALS-SimVR was built using the Unreal game engine [38]. This choice was made as it is considered to have textures and editing capacity and provides a finished product of a high visual standard. At the heart of the ALS-SimVR app is the replication of the complex decision-making processes. The actions are broken down into small segments of code that populate decision trees. Using Unreal visual scripting, these actions and descriptions were formed into nodes. These nodes were then compiled together to make blueprints that convert user decisions into game-based actions. The use of blueprints allows for actions containing code, audio, animation, and facial motion capture to easily be used in other sequences and scenarios. Running concurrently with the application were analytics that recorded all decisions and interactions. These analytics allow for the generation of the user feedback document provided upon the completion of the scenario. The analytics also populated the scribe notes, which were available for reference upon user request during the scenario. This is a vital resource utilized by clinicians during ALS events.

## Methods

In total, 10 clinicians with ALS experience in both clinical and educational settings were recruited to provide expert input. The research was approved by the University of Sydney Human Ethics Committee (2016/089). All participants completed written informed consent forms.

At the start of the experiment, participants were given a brief overview of the study and instructed on how to use the Oculus GO headset. Participants then performed a playthrough of the cardiac arrest scenario and managed the patient as they would in a real clinical setting. Their sessions were recorded using the Oculus livestream feature and ran for approximately 10 minutes.

Upon completion of the scenario, a semistructured interview was conducted, which was audio-recorded with the participant’s permission. Each interview lasted approximately 20 minutes. Participants were first asked about their immediate overall impression and experience. Participants were then asked about the usability of the application and their own performance. These usability questions asked the participant for their thoughts about the 12 heuristics items in Sutcliffe and Gault's heuristic evaluation of VR apps [39], which were read out to the participant to ensure a consistent but broad coverage of specific performance aspects. If the participant’s response was vague, the interviewer asked for further clarity. The heuristic items are the following: natural engagement, compatibility with the use’s tasks and domain, natural expression of action, close coordination of actions and representation, realistic feedback, faithful viewpoints, navigation and orientation support, clear entry and exit points, consistent departures, support for learning, clear turn-taking, and sense of presence. Next, the participants rated the severity of performance issues relevant to each item on a 4-point scale (Severe, Annoying, Distracting, or Inconvenient) as per the standard questionnaire [39].

Finally, participants provided any further comments before completing the Université du Québec en Outaouais Revised Presence Questionnaire (PQ) with 24 items, each rated on a 7-point scale [40]. In the context of this study, we excluded items 23 and 24 as they were concerned with haptic experiences that did not apply to ALS-SimVR. The 22 remaining items are classified into 7 presence factors in the PQ: realism, possibility to act, quality of interface, possibility to examine, self-evaluation of performance, sounds, and haptics. Each item is formulated as a question (eg, How much were you able to control events?) with the 7-point scale ranging from the lowest score (1=Not at all) to the highest (7=Completely).

## Results

### Overview

Participants (n=6 female, n=4 male) had an average age of 40.9 (SD 9.48) years, and an average of 12.4 (SD 6.93) years of experience providing or teaching ALS. All participants had minimal to no previous experience in using VR in either a personal or education capacity. All audio interviews were transcribed by a blinded researcher. Two researchers (one expert in teaching ALS in the clinical setting and another a human-computer interaction researcher) worked together and followed an inductive (or bottom-up) approach to the analysis of qualitative feedback received from participants [41]. No assumptions were made about a coding system and instead participant statements were clustered based on similarity in content, after which higher level themes were identified and labelled. The analysis aimed to identify the needs and expectations of the participants relative to their experience with the ALS-SimVR. The average ratings for usability and presence were then calculated to reinforce the statements provided in the interviews.

### Interviews

A number of insights were derived from the participant statements, which were classified into five themes summarized in Table 1, supplemented with brief descriptions and subthemes when relevant. Each of the five themes are discussed next.

**Table 1:** User needs groupings and exemplar statements.

User needs theme		Exemplar statements
**Affordances**		
	Realistic tasks	Common clinical tasks should be available for completion in a realistic manner
	Visibility	Clear visible assets aligned with environmental orientation
	Completion	Clear commencement and completion prompt to task
	Accessibility	Clarity as to how commands are given and accessed
Agency		The environment providing opportunity to control workflows autonomously and make choices that align with prior experiences, such as multitasking
Diverse input modalities		The environment replicates natural input modalities such as issuing commands verbally
Mental models		The environment design and prompts align with how the clinical environment operates (eg, 2 minutes of cardiopulmonary resuscitation completion)
Advanced roles		The ability to manage tasks at an acceptable clinical standard

### Affordances

#### Overview

Affordances often represent relational approaches to the way people interact with technologies [42]. In relation to ALS-SimVR, we specify affordances as users’ concern for the availability of action within the virtual environment. The participants in our study noted four specific affordances that needed to be addressed through design. These are detailed below.

#### Realistic Tasks

Participants identified a need for the ability to do the tasks in a virtual environment in a realistic manner, replicating the clinical environment. Although the app was designed to recreate the majority of the initial requirements for ALS management, some participants (in particular, more senior clinicians) were not able to complete the tasks in a manner that mirrored real clinical management. For example, the programming constraint in the ALS-SimVR did not allow the participants to return to their previous decisions and change what they had previously done, which is typical in patient management.

#### Visibility

Participants highlighted the need for visibility of actors and objects they needed to interact with in the virtual environment. For instance, the positioning of the nurse scribe avatar within the virtual environment was visually inaccessible to the participants (the avatar was slightly behind their right shoulder) and it took a while for participants to notice the scribe was there. This resulted in some participants missing out on some of the functionality of the app. However, once the participants had oriented themselves, they reported the positioning of the team members accessible and “good.” A design recommendation emerging from this need is to allow more time within future solutions of this type to allow users to explore their surroundings prior to commencing a scenario. Another example of visibility issues that emerged in the study was that the three degrees of freedom and resolution limits of the VR headset resulted in some participants being unable to read or clearly visualize some assets within the environment.

#### Completion

Knowing when all tasks were completed and the scenario had actually ended was an affordance issue identified by some participants. Upon completion of the scenario, the participant was presented with a large report document within the app titled “Results.” This provided them with feedback on their performance, including compliance with the ALS algorithm, defibrillation, and intervention accuracy. The report appeared in the center of their vision, between the patient and the participant. Some of the participants chose to immediately close the document without reading it and continued with the scenario. Due to the design of the app, they were not able to find and review that document at a later time, which seemed to negatively impact their experience.

#### Accessibility

Participants highlighted the need for greater orientation in how to start “playing the game.” One participant commented “I needed guidance to know how to play the game.” Additionally, participants wanted clarity around some design elements that they could not identify affordances for. For instance, participants did not realize how an interactive text box provided the available interactions or tasks and how they could reopen them. Participants expressed their preferences for interface accessibility including readability of design elements (eg, larger text size and clickable elements), visibility of design elements (eg, a more comfortable distance to a pathology report that was positioned too close), or flexibility of actions (eg, flipping through a chart). These were departures from the realities of managing patients in the clinical setting and were consequently deemed “annoying” by participants.

### Agency

Participants wanted the opportunity and control to do things autonomously, based on their personal experiences. In the context of health care, this poses challenges as the complex nature of managing unwell patients means clinicians make different choices depending on their seniority, experience, and education. This requires a level of agency that was not supported in ALS-SimVR. Additionally, having the agency to make such choices may require multitasking, which the app did not enable. Participants wanted the capacity to plan ahead and even allocate tasks to different team members.

### Diverse Input Modalities

Participants discussed their desire for a diverse set of inputs to the device, similar to real life, for instance by using voice commands in addition to using the hand controller. One participant stated, “I just didn’t like not being able to talk” and “I wanted to talk to the team to ask for updates.” This particular activity seemed divisive among participants with some expressing an absolute need for it to reflect the real nature of work and others stating this was not a problem for them.

### Mental Models

Overall, 3 participants identified instances where the actions they took within the virtual environment did not align with the approach they would take in the clinical environment (ie, their preconceived mental models). Clinical decisions made within the ALS-SimVR are recorded and analyzed to form an assessment of user performance at the completion of the scenario. The way this is programmed within the app meant that once those decisions are made, they cannot be undone or revisited. This created a disconnect for a number of participants whose mental models or real-world practice would entail frequent reassessment of their patient and decisions to escalate the situation accordingly.

### Advanced Roles

A number of additional roles in the ALS-SimVR scenario were suggested by the participants to create new interpersonal interactions within the app, for example to access the patient assessment report by asking someone on the team instead of clicking on the patient. One participant noted that the scenario “didn’t have extra people.” Another reported the “need to practice with team members not knowing their roles perfectly” is a constant challenge for real-life implementation of this training. Other suggestions included advanced roles (through avatars) for cardiopulmonary resuscitation and to demonstrate listening to the chest.

### Usability Heuristics

The participants rated the severity of each heuristic item [39]. Two items (Item 7 – Navigation and orientation support, and Item 11 – Clear turn taking) were eliminated from the analysis as they were not relevant to this application. The ALS-SimVR is a single player experience that did not allow the user to move in their environment and did not involve turn taking. The results of these ratings are summarized in Table 2. The number of issues reported for each item and their corresponding severity was calculated. For instance, for the first item (natural engagement), we received 2 statements implying a “severe” issue, 5 statements implying an “annoying” issue, 8 statements implying “distracting” issues, and 11 statements implying “inconvenient” issues. A total score was then calculated for each item by adding the total number of reported issues.

Overall, 3 items clearly had the highest usability issues: Item 1 – Natural engagement (with a total score of 26), Item 3 – Natural expression of action (with a total score of 31), and Item 12 – Sense of presence (with a total score of 15). This is an indication of areas of priority for improving the usability of ALS-SimVR.

**Table 2:** Results of heuristic responses, noting the number of statements relevant to each item and the corresponding severity ratings.

Heuristic item	Severity rating				Total/item	
	Severe	Annoying	Distracting	Inconvenient		
Natural engagement	2	5	8	11		26
Compatibility with users’ task and domain	1	—^a^	3	5		9
Natural expression of action	2	3	15	11		31
Close coordination of action and representation	—	—	2	1		3
Realistic feedback	—	—	—	2		2
Faithful viewpoints	1	1	—	2		4
Navigation and orientation support	—	—	—	—		N/A^b^
Clear entry and exit points	—	3	1	3		7
Consistent departures	—	-	1	1		2
Support for learning	1	2	3	5		11
Clear turn taking	—	—	—	—		N/A
Sense of presence	1	—	4	10		15

^a^Not available.

^b^N/A: not applicable.

### Presence

The participant responses to the relevant 22 items on the PQ were compiled. The standard scoring system classifies the responses into 7 themes: realism, possibility to act, quality of interface, possibility to examine, self-evaluation of performance, sounds, and haptic (not present in this study). The rating scores received for all items relevant to each theme were averaged and then summed. The average rating scores and standard deviations of the 7 themes are presented in Table 3.

The findings suggest realism received the highest score (average score 35, SD 10.1), indicating the success of ALS-SimVR in replicating a realistic experience and potentially resulting in an enhanced sense of presence in the virtual environment. Conversely, self-evaluation of performance received the lowest score (average score 10.6, SD 3.3), indicating a poor experience of self-evaluation, which diminishes the participant’s sense of presence in the virtual environment.

**Table 3:** The average rating scores on the 7 Presence Questionnaire items indicate the participants’ experience of their presence in ALS-SimVR, a virtual reality Advanced Life Support training application.

Presence Questionnaire item	Average (SD)
Realism	35.0 (10.1)
Possibility to act	21.9 (5.6)
Quality of interface	13.1 (5.0)
Possibility to examine	14.0 (3.9)
Self-evaluation of performance	10.6 (3.3)
Sounds	16.5 (5.4)
Haptics	N/A^a^

^a^N/A: not applicable.

## Discussion

### Principal Findings

Our findings provide insights as to how VR applications could be improved to better address the needs of clinicians in the context of ALS training. There is evidence that the use of virtual patients can be beneficial to increasing the engagement of clinicians [43]; however, the evidence on how that can be achieved is limited. Our study explored specific user needs to improve their engagement with the interactive simulation training app. We conducted usability testing and interviews with 10 expert participants with experience in ALS. Their feedback revealed important consideration for the ALS-SimVR app in five categories that stipulate how VR training should be situated to support the learning experience: affordances, agency, diverse input modalities, mental models, and advanced roles. These provide detailed design directions for improving ALS-SimVR and developing any application of this type in the future.

The usability testing revealed higher scores for natural expression of action, natural engagement, and sense of presence, indicating areas of improvement. Lower scores for usability items such as consistent departures, realistic feedback, as well as close coordination of action, representation, and faithful viewpoints, indicate areas in which ALS-SimVR performed well. The findings from the PQ complemented, gave scope and, in some instances, reinforced some of the findings of the heuristic-based usability testing and uncovered additional user perspectives. For instance, realism and possibility of actions were scored high in the PQ, which can be attributed to the natural engagement and expression of actions mentioned in the usability tests. These are discussed further in this section. The interview findings revealed important avenues for further investigation into the design of future applications in this field, especially in relation to what affordances should be prioritized in design and potential challenges to user engagement.

### Clinical Training Through VR: Situating Affordances in the Learning Experience

It is clear from our findings that any VR application for health care training must capture accurate and realistic affordances. Previous evidence linked realism to successful VR training, for instance in surgical training [44]. Our assessment of the ALS-SimVR yielded mixed results.

On the one hand, realism was scored high in the PQ but on the other hand, a relatively high number of presence issues were identified through the heuristic usability testing, with most issues linked to natural expression of actions. Clear opportunities for future development, based on collected feedback, pointed to the need for clarity and accessibility in problem-based learning [45], which underlined the training experience in ALS-SimVR.

The clinicians who participated in our study desired an educational package that allows them to function as they would in the clinical environment in a high-fidelity, realistic, and engaging way. This remains an ongoing challenge in the design of VR-based health care training simulations [19] due to the complex management choices and decision making required to care for deteriorating patients. Our findings suggest clear directions for addressing some of these challenges.

We propose that designing for realistic affordances should focus on “realistic actions” afforded to trainees in the virtual space. For instance, the interactive VR simulation should allow users to perform tasks in a realistic manner, to have good visibility, a clear beginning and end to tasks, and the ability to give commands. The latter, in our experience, is more challenging to achieve. This is also clear from the notable number of issues relevant to natural engagement (see Table 2), as our participants demanded the ability to walk around the virtual environment. This mirrors real-world ALS training where, although the team leader stands at the foot of the patient bed, they often move around to get a better view. This was not afforded by the ALS-SimVR hardware, which at the time only offered three degrees of movement.

### Challenges and Opportunities in Interactive Medical VR Simulations

The thematic analysis of the interview results highlighted interaction challenges for users. Our findings suggest that diversifying the input modalities can be a remarkable opportunity to improve the user experience in interactive VR simulations. This is evident from the low score given to the sound item on the PQ (Table 3). A contentious issue among participants was the lack of voice control. A number of participants stated that voice control would not be necessary because the visual communication through the text boxes within the app was clear and worked for them, and they felt they could easily “fill in the gaps.” Other participants, however, desired the opportunity to use voice as an input modality.

While this was an important issue, the literature provides limited discussion around it. Addressing diversity in input modalities has become a challenge the research team has devoted significant time to but has not yet overcome. Voice control was part of the initial design plan for the ALS-SimVR due to an indication from other health care settings [46]; however, implementing it is not without significant challenges. In addition to the computational and algorithmic challenges, there is the issue of a hugely diverse cohort of individuals, all of whom often use different language to convey the same information using expressions drawn from a diverse and specific lexicon [47].

As a result, we propose other considerations for diversifying input modalities, such as the use of haptic inputs or hand gestures. The use of simple Oculus Go hand controllers in this study was met with mixed opinions by the participants due to the limited number of actions supported by the controller. The link between the choice of technology (or controllers) and simulation experience in medical training is not trivial, and yet lacking in the literature. Although our participants indicated a preference for diverse modalities that were not supported through the simple interactions offered by Oculus Go, our observation reveals the device did not interfere greatly with the participant’s ability to perform the required tasks within ALS-SimVR. One explanation for this could be that whilst there were some limitations in the design of the ALS-SimVR app, many of the actions and interactions required were still achievable. However, the heuristics usability testing is designed to identify “issues” rather than highlight the positives, which may have led to this emphasis on the lack of diverse modalities. Further investigation into this matter is therefore warranted in future research.

Diversifying input modalities can potentially impact the experience of agency [48]. Agency in VR is generally defined as the experience of controlling actions and their consequences in the virtual environment [48]. Previous research has illustrated that the sense of agency in VR is diminished when relying on voice commands instead of hand controllers [48]. Therefore, the challenge with using voice in VR apps similar to ALS-SimVR is to combine it with other modalities that are meaningful to users and give them a sense of control. Our exploratory study did not address this issue. Further research is needed to identify which design features can improve the sense of agency in VR clinical training apps.

We found that assessing the experience of agency is particularly challenging. Agency is not directly addressed through the questionnaires we used in our research, but there are a number of heuristic items that may be linked to the experience of agency in the context of ALS-SimVR. For instance, support for learning, ability to coordinate actions, and compatibility of the app with the user’s task and domain. These items align with the definition of agency as the ability to control actions. Additionally, the results from the PQ indicated the satisfaction of our participants with the “possibility to act”, which can be interpreted as closely linked to agency. We propose that future research should specifically examine effective ways of evaluating agency in interactive VR experiences in medical training.

### Limitations

A number of design and methodological limitations in our study warrant further research. Enabling clinicians to change their mind or edit decisions in ALS-SimVR posed challenges for the designers. As soon as any user choice is made in ALS-SimVR, it is logged and recorded to the database to generate feedback during the game and compile the “result” report at the end. The database was not designed to be edited once a decision was made, which means less flexibility of actions for users. Creating an updatable database requires more powerful hardware processing capability and continuous reviewing. It would also require a lot more coding development to allow the interruption of ongoing animations triggered by previous decisions. These were outside the available resources for the initial ALS-SimVR prototype used in this study.

From the methodological perspective, we intended to conduct an in-depth study; however, our sample size is small and from a clinically nondiverse background and included participants with little to no VR experience. As such, we received comments regarding application orientation that may not be applicable to more experienced users. Lack of VR experience made the learning curve of the app more challenging for our participants, as they had to familiarize themselves with a new technology. In addition, the prototype did not have an in-built orientation tutorial and the participants were given a brief verbal orientation. Additionally, with the high level of clinical expertise of the study group, there is a potential bias from the high level of clinical performance within the application. Future research should address these limitations. The findings from our qualitative study should be further validated through more testing and quantitative methods should be used to objectively address the five proposed themes.

### ALS-SimVR: Next Steps

Previous research had suggested that high-fidelity interactive training simulations can foster clinician confidence [49]. However, in our study, new questions emerged on the effects of fidelity and realism within the immersive virtual clinical training environments. Questions targeting the realism of ALS-SimVR concern participant engagement and reflection on lived real-world experiences and underline the need for more immersive visuals and audio. The orderly and “sterile” nature of the current scenario detracted from the overall realism of the experience for users as it did not align with the common experience of clinicians responding to these events. This perceived sterility also leads us to believe there is further scope to explore the role of mental models and advanced roles within the ALS-SimVR.

The responsibility of being the team leader in an ALS scenario requires establishing and implementing mental models with a high degree of awareness toward potential consequences. For the effective management of a cardiac arrest or significant clinical deterioration, a team leader must be able to preplan their actions [50]. The ALS-SimVR app did not provide the opportunity for clinicians to demonstrate this preplanning. The linear nature of the decision-making process within the app and inability to stack or preset actions throughout the scenario distracted some users. A future iteration of ALS-SimVR will need to support this type of processing.

ALS is an advanced skill set requiring advanced roles and interventions [51]. The findings of this study highlighted that the challenge of providing users with the ability to perform these interventions must be considered and ideally overcome to maintain engagement. Our participants frequently brought up the importance of practicing advanced roles during interviews and this will inform future iterations of ALS-SimVR.

Since the completion of this study, a number of these identified issues have been addressed and included in subsequent versions of the application [52]. The application now runs on the Oculus Quest headset, which allows for greater processing power, higher resolution, and six degrees of freedom [53]. ALS-SimVR now has randomization of events and results to allow for greater replay ability, user performance is recorded and available for their own viewing and reflection on a web portal, and a tutorial has been added to standardize the introduction to the application. Finally, the animations and visualizations have also been improved.

Moving forward, the research team will gather additional data using other iterations of the application to further guide design and development for this application and VR-based educational design in general.

### Conclusion

Interactive VR simulations and gameplays are increasingly used to augment traditional methods of clinical training such as ALS. However, there is limited information about how design can support the user experience and engagement needs in such VR apps. This study presents user-centered research on ALS-SimVR, an application designed to deliver ALS training. Based on interviews and usability testing of ALS-SimVR with 10 experienced clinicians, we identified five main areas of design considerations which should be considered when developing VR applications in this context: affordances, agency, diverse input modalities, mental models, and advanced roles. It was determined that a desirable user experience that supports ALS training and learning needs in VR must afford autonomous interactions within the VR app to allow users to complete the required clinical actions and make decisions in a way that is familiar to them and replicates real life. Diversifying input modalities to include voice commands within the VR environment may enhance the trainees’ experience of realism; however, this must be approached with care and combined with other modalities. Additionally, the tasks in VR must align with clinicians’ mental models for the role they are assigned in the game and how they would normally approach that challenge (eg, treating a patient). This may require careful consideration of interactions with other advanced roles in VR. The scale and spectrum of the interventions possible in this setting can provide challenges in creating a meaningful and realistic experience. The insights generated in this study can inform the design and testing of other similar VR-based training applications in health care, which is an area that requires further research [54].

## Abbreviations

ACLS: Advanced Cardiac Life Support
ALS: Advanced Life Support
ALS-SimVR: Advanced Life Support Simulation in VR
HMD: head-mounted devices
PQ: Presence Questionnaire
VE: virtual environments
VR: virtual reality
